# Two Cases of Anterior Shoulder Dislocation and Fracture Secondary to Generalized Tonic-Clonic Seizure

**DOI:** 10.1155/2024/6652622

**Published:** 2024-02-07

**Authors:** Kevin Trong Dao, Hari Kunhi Prasad Veedu, Britney Ly, Neela Zalmay, Rajashree Hariprasad, Michael Eagan, Najib Ussef

**Affiliations:** ^1^Department of Medicine, Division of Neurology, Kern Medical-UCLA, Bakersfield, CA, USA; ^2^Department of Surgery, Division of Orthopedic Surgery, Kern Medical-UCLA, Bakersfield, CA, USA

## Abstract

Dislocation of the glenohumeral joint secondary to generalized tonic-clonic seizures is well documented in the medical literature, with posterior dislocation being most commonly described. Still, these occurrences tend to be rare and affect a minority of patients, and fractures associated with dislocations after seizures are even less common. As such, the management of these injuries tends to be quite varied, and there is a paucity of documented cases in the literature. Here, we would like to present two rare cases of anterior shoulder dislocation secondary to seizures, with one patient also sustaining a fracture of the proximal humerus. We would also like to discuss the management and outcomes that have been achieved, since these cases tend to occur in a small number of epileptic patients.

## 1. Introduction

Shoulder dislocations generally account for approximately 45% of all dislocations seen in the emergency department [1], with anterior dislocations being the most common. Posterior dislocations, on the other hand, are quite rare and generally account for only about 5% of shoulder dislocations [2]. This is true in patients who have traumatic shoulder dislocations, where posterior shoulder dislocations occur at a rate of just 1.1 per 100,000 people per year [3]. In epilepsy patients, however, posterior dislocations occur more commonly due to a distinct mechanism whereby seizure activity causes powerful muscular contractions in the shoulder girdle. When this is combined with axial loading of the glenohumeral joint with the shoulder in an adducted and internally rotated position, posterior dislocation of the joint may occur [4]. A similar mechanism may also occur during accidental electrocution or from lightning strikes, making these patients the second group that has been classically described as being at risk of posterior dislocation [5]. Another study found that recurrent dislocations could be caused by bone loss from the humeral head and glenoid as a result of previous seizure-associated dislocations [6]. Still, seizure-related dislocations are not common. In fact, shoulder dislocations occur in just 0.6% of seizure patients, with even fewer resulting in concomitant fractures of the shoulder region [6, 7]. When shoulder dislocations do occur as a result of a seizure, the most common presentation is a bilateral posterior shoulder dislocation, occurring in 33% of patients [8]. Since these seizure-induced dislocations are uncommon, we would like to present a case series involving two patients, both of whom had an anterior shoulder dislocation secondary to a seizure, with one patient having an associated ipsilateral proximal humerus fracture. A discussion of medical and surgical management will also be included.

## 2. Case 1 Presentation

A 27-year-old male with refractory nonlesional bitemporal lobe epilepsy since late 2017 presented to the clinic to establish care. He noted that he had a history of recurrent left patella and right shoulder dislocations associated with his seizures. The patient had sustained a total of sixteen shoulder dislocations, with the majority of them attributed to his convulsions between 2017 and 2021. A few occurred independently without seizures. He is currently taking levetiracetam and lamotrigine and denies taking any other medications. On chart review from a prior facility, the patient had a history of both seizures and pseudoseizures.

**Figure 1:**
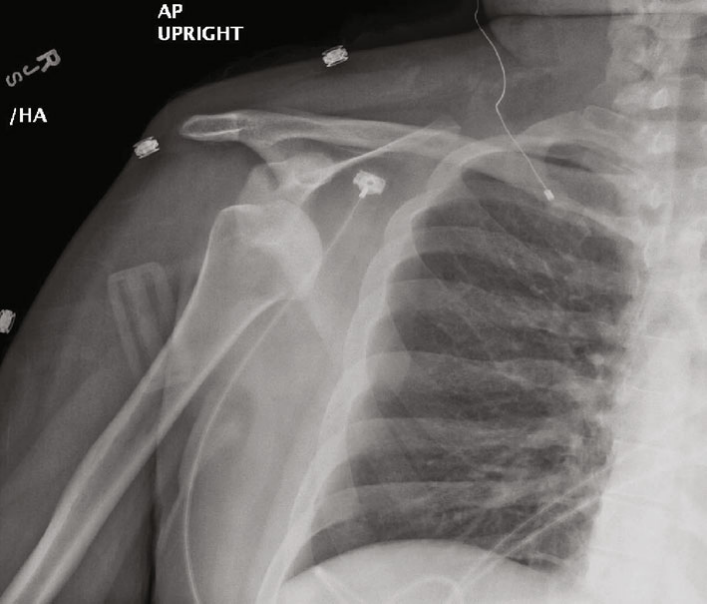
AP right shoulder, prereduction (case 1). Typical radiographic appearance of an anterior glenohumeral dislocation.

**Figure 2:**
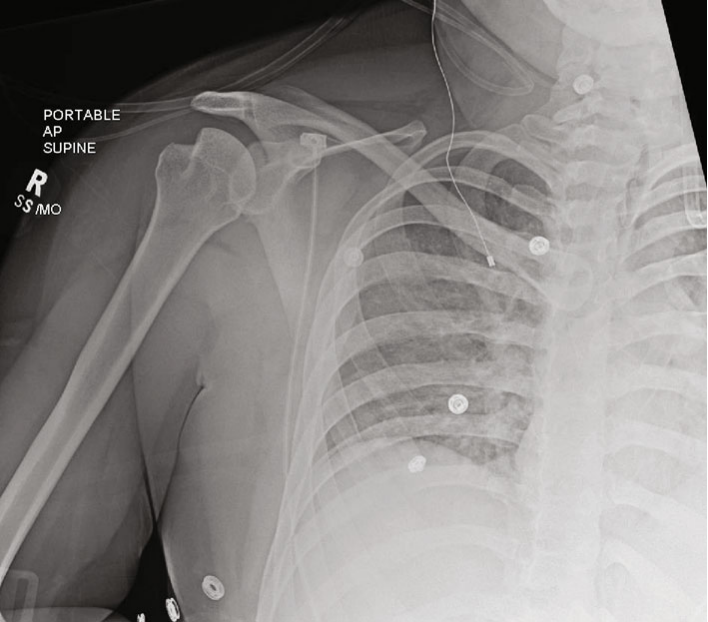
AP right shoulder, postreduction (case 1). There is successful reduction of the glenohumeral joint. Prominent Hill-Sachs lesion of the humeral head is noted. Poorly defined bony fragments are barely visible along the inferior glenoid.

**Figure 3:**
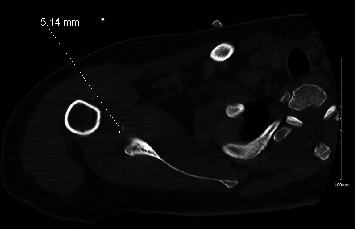
Axial CT scan, right shoulder, postreduction (case 1). The anterior shoulder dislocation has been reduced, and multiple bony fragments measuring up to 5 mm are seen adjacent to the anterior inferior glenoid, representing a bony-Bankart lesion.

**Figure 4:**
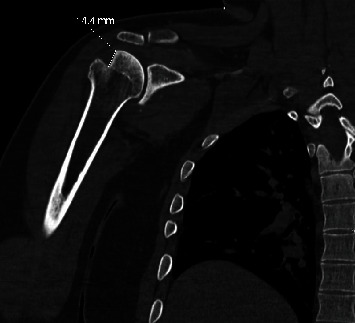
Coronal CT scan, right shoulder, postreduction (case 1). The large Hill-Sachs lesion of the humeral head is clearly seen, with nearly 15 mm of bony impaction.

As a part of the epilepsy workup, he underwent an epilepsy monitoring unit (EMU) evaluation. Throughout the course of the EMU evaluation, the patient's antiepileptic medications were slowly tapered and stopped. On the 7th day of the EMU stay, he had one of his habitual electroclinical seizures, which was classified as a focal to bilateral tonic-clonic seizure associated with right temporal lobe EEG onset. The patient sustained a right shoulder dislocation during the convulsive seizure. Shoulder radiographs demonstrated an anterior glenohumeral dislocation (Figure 1). Orthopedic consultation was requested; a closed reduction of the shoulder was performed; and the patient was placed in a sling. Postreduction X-rays showed abnormalities of the inferior glenoid and humeral head, and a dedicated shoulder CT scan was ordered (Figure 2). A CT scan confirmed a Hill-Sachs lesion in the humeral head and a bony-Bankart fracture of the anterior/inferior glenoid (Figures 3 and 4). The diameter of the inferior glenoid was approximately 30 mm, while the width of the anterior glenoid bone loss was close to 6 mm. The patient's Hill-Sachs lesion was measured to be roughly 20 mm, which was greater than the glenoid tract calculation (0.83 (30 mm) − 6 mm = 18.9 mm) meaning that this patient's shoulder is “off track” [9]. As such, orthopedics suggested surgical intervention to minimize the risk of recurrent instability and requested a neurology evaluation before proceeding.

The patient's detailed epilepsy presurgical workup showed that the patient has nonlesional bitemporal lobe epilepsy and is waiting to undergo bilateral responsive neurostimulation (RNS) implantation to control his epilepsy. Orthopedics recommended that the patient remain seizure free for at least 3 months before proceeding with surgery for shoulder stabilization.

## 3. Case 2 Presentation

A 34-year-old male with a history of traumatic brain injuries presented to the emergency department complaining of left shoulder pain after waking up confused. His traumatic brain injury occurred in 2006 when he fell from a tree and sustained fractures of the left radius and ulna, along with concussions of the brain and cerebral edema. The patient's first seizure occurred in 2014, during which time he also sustained a left shoulder dislocation that was treated with closed reduction. However, he was not started on antiseizure medications at the time.

**Figure 5:**
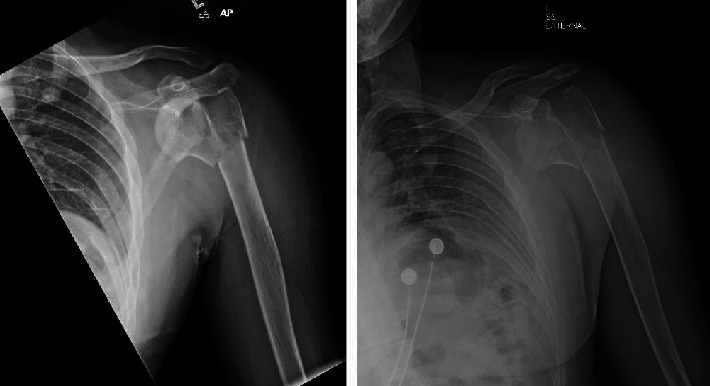
AP left shoulder, preop (case 2). A comminuted fracture deformity of the left humeral neck and greater tuberosity with dislocation of the glenohumeral joint.

**Figure 6:**
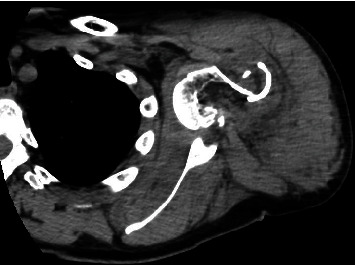
Axial CT scan, left shoulder (case 2). Comminuted fracture of the proximal humerus with complete anterior dislocation of the glenohumeral joint.

**Figure 7:**
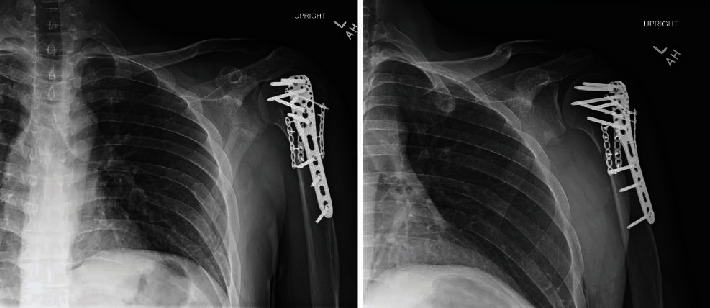
AP left shoulder, postop (case 2). The proximal humerus fracture has been reduced and repaired using multiple plates and screws. The glenohumeral dislocation has been successfully reduced.

In the emergency department, a physical exam revealed restricted shoulder motion secondary to pain and guarding. The left shoulder X-ray was noted to have a significantly displaced fracture of the proximal humerus with dislocation of the glenohumeral joint (Figure 5). The CT shoulder showed a comminuted proximal humerus fracture with anterior dislocation of the glenohumeral joint and a bony-Bankart fracture of the anterior inferior glenoid rim involving about 20% of the articular surface area (Figure 6). Neurology was consulted, and the patient was started on antiseizure medications, namely, levetiracetam. The patient underwent open reduction and internal fixation of the proximal humerus fracture as well as open reduction of the glenohumeral joint (Figure 7). He was noted to have residual anterior instability after internal fixation with the shoulder in external rotation and was placed in a shoulder immobilizer (UltraSling) with the shoulder internally rotated.

**Figure 8:**
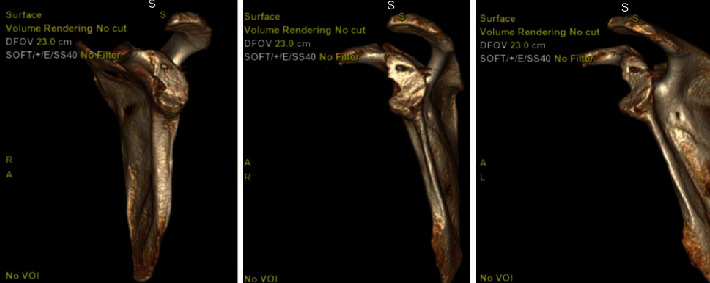
CT scan left shoulder with 3D reconstruction and humeral subtraction (case 2). A large glenoid rim fracture fragment is seen involving approximately 20% of the articular surface.

**Figure 9:**
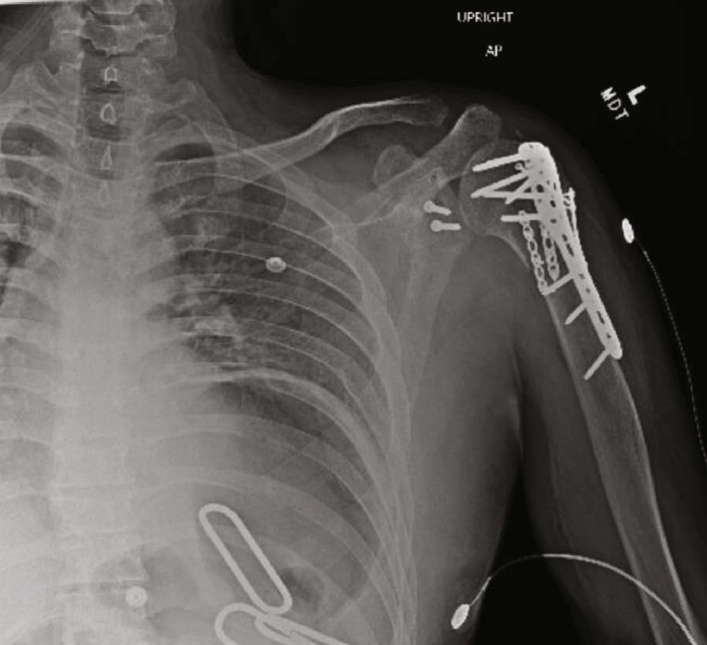
AP left shoulder, post-Latarjet (case 2). The coracoid process has been transferred to the glenoid and stabilized with 2 screws.

Postoperative CT scan of the shoulder was performed to confirm appropriate reduction of the proximal humerus fracture and glenohumeral joint, and the glenoid deficiency was again evaluated. The glenoid bone loss was calculated via the “best fit circle technique” [10], which was calculated to be 25%. No off track/on track calculation was made given the proximal humerus fracture. The patient elected to proceed with a shoulder stabilization procedure to minimize the risk of recurrent dislocation, and options were discussed. In this patient, instability resulted from a defect in the anterior/inferior glenoid, so stabilization would focus on restoring bone loss in that region (Figure 8). In some instances, where the fracture is a large fragment without comminution, internal fixation of the fragment may be possible and sufficient to prevent recurrence [11–16]. In instances of recurrent dislocation where glenoid bone loss is more severe or the fragment is comminuted or not amenable to fixation, other options include bone grafting of the defect using autograft or allograft bone or, as in this case, the Latarjet procedure. In this patient's case, a distal tibial allograft was at the forefront of consideration; however, due to limited resources in underserved setting, no graft could be performed, and the Latarjet was the best alternative. In a Latarjet procedure [11–16], the anterior glenoid is exposed via an anterior surgical approach, and the coracoid process of the scapula is exposed along with the muscular origins of the coracobrachialis, the pectoralis minor, and the short head of the biceps. Osteotomy of the tip of the coracoid process is performed, and it is transferred laterally and inferiorly to the glenoid defect and stabilized with screws. The shoulder is protected with limited weight-bearing and motion to allow the bone to incorporate into the glenoid defect, and shoulder rehabilitation is instituted to regain range of motion and strength. The patient tolerated the procedure well and, on the most recent follow-up, was recovering and doing well in physical therapy without recurrent dislocation (Figure 9). On neurology follow-up, he denied seizure recurrence since discharge from the hospital.

## 4. Discussion

Shoulder dislocations due to epilepsy tend to be rare events and are frequently bilateral, with one study noting only 12 cases of bilateral anterior shoulder dislocation [17] and another noting less than 40 cases of bilateral posterior fractures or dislocations [18]. There appear to be fewer documented cases of unilateral shoulder dislocations, but this does not mean that they should be overlooked.

This case also raises the question of EMU safety measures to be followed in patients undergoing continuous video EEG monitoring as a part of the epilepsy surgery workup. Although EMUs are considered a crucial diagnostic tool to diagnose epilepsy, the benefits should outweigh any expected risks. In case 1 presentation, the patient failed multiple antiseizure medications prior to EMU admission. Thus, we felt that an EMU evaluation was necessary prior to consideration for epilepsy surgery or neuromodulation implantation to control his seizures since the patient is only cleared to undergo shoulder stabilization surgery under the contingency that he is seizure free for at least 3 months. The patient was informed of possible risks regarding recurrent shoulder dislocation, and anticipatory precautions were taken in the event of shoulder dislocation. This complication highlights one of the less common but possible risks that should be discussed with the patient prior to proceeding with an EMU study. Appropriate precautions should be in place to protect patients in the event of shoulder dislocation, and access to multidisciplinary care is important to manage any complications that may result. In both of our patients, orthopedic consultations were available to manage the shoulder dislocation as needed.

The first step in managing a possible shoulder dislocation is the evaluation of the patient, which includes a neurologic examination of the extremity, especially focused on the brachial plexus and the axillary, median, ulnar, and radial nerves. The most commonly encountered nerve injury following a dislocation is the axillary nerve, and this can be assessed by checking for sensation over the deltoid muscle or by testing for deltoid muscle contraction. The next step is imaging the shoulder. Plain radiographs are generally adequate to diagnose major fractures, but smaller fractures of the glenoid or humerus may not be adequately seen. If possible, high-quality images of the shoulder should include AP, scapular-Y, and axillary views, although these can often be difficult or impossible to obtain because a patient in pain cannot appropriately position the arm. Furthermore, subtle anterior or posterior subluxation of the glenohumeral joint is difficult to assess on even the highest quality plain X-rays. If the X-rays are questionable or inadequate, a CT scan should be performed. When a diagnosis of acute dislocation is made, the shoulder should be reduced as soon as possible. Depending on the resources available, this may be done in an emergency room or at the bedside in some cases. In cases of an associated fracture, closed reduction may not be possible, and open reduction may be necessary. Consultation with an orthopedic surgeon is recommended, and treatment can vary substantially based on the character of the injury. In patients who undergo closed reduction, a postreduction CT scan may be recommended to confirm reduction and evaluate fractures, or for preoperative planning. MRI is also recommended in most cases to evaluate for other pathologies such as anterior or inferior glenoid labrum tears (Bankart lesions), humeral head injuries (Hill-Sachs lesions), rotator cuff tears, etc. Definitive treatment can range from simple nonoperative management in a sling followed by physical therapy, to shoulder arthroscopy and labral repair or rotator cuff repair, to arthroscopic or open stabilization using bone grafting or Latarjet [2, 6, 15, 17–20]. Complications of dislocation and surgical intervention can include neurologic injury, shoulder stiffness or loss of motion, avascular necrosis [15, 18], degenerative joint disease, and recurrent instability. Antiepileptic treatment should be initiated to ensure no further seizure activity, and close follow-up is highly recommended.

## 5. Conclusion

With 5 million people being diagnosed with epilepsy every year, it is crucial that physicians remain vigilant to minimize seizure-related complications. As such, a thorough history and physical exam should be performed to rule out musculoskeletal and neurological injuries after a seizure, and liberal use of imaging is highly recommended in patients with new musculoskeletal complaints after a seizure. In patients with a reported history of seizures who are taking antiepileptic medication, EMU studies can be considered since they are an important diagnostic tool to understand the evolution and development of seizure activity [21]. However, these are not without risk, and it is important to inform the patient of the risks and benefits. In both of our cases, a history, physical exam, and imaging were important factors in making a diagnosis, and treatment generally consisted of antiepileptic medications and orthopedic consultation. Orthopedic intervention may vary widely because of the heterogeneous nature of the injuries, but treatment may be operative or nonoperative and may include internal fixation or a multitude of strategies aimed at restoring stability to an unstable joint [2, 6, 15, 17–20].

Physicians should be aware that patients with a history of epilepsy or seizures are somewhat more prone to dislocations and fractures of the shoulder and may develop joint laxity, although the incidence is still quite low [3, 8]. Understanding this cause and effect is important to identify patients at risk and provide better care to patients with epilepsy.
